# The *Escherichia coli*-Derived Thymosin **β**4 Concatemer Promotes Cell Proliferation and Healing Wound in Mice

**DOI:** 10.1155/2013/241721

**Published:** 2013-05-19

**Authors:** Xiaolei Wang, Guihua Yang, Shanshuang Li, Meifeng Gao, Pangfeng Zhao, Lingxia Zhao

**Affiliations:** Plant Biotechnology Research Center, School of Agriculture and Biology, Fudan-SJTU-Nottingham, Plant Biotechnology R and D Center, Shanghai Jiao Tong University, Shanghai 200240, China

## Abstract

Thymosin **β**4 (T**β**4) is one of the most promising thymosins for future clinical applications, and it is anticipated that commercial demand for T**β**4 will increase. In order to develop a new approach to produce recombinant T**β**4, a 168 bp DNA (termed *T*β**4) was designed based on the T**β**4 protein sequence and used to express a 4 × *T*β**4 concatemer (four tandem copies of T**β**4, termed 4 × *T* **β**4) together with a histidine tag (6 × His) in *E. coli* (strain BL21). SDS-PAGE and western blot analysis were used to confirm that a recombinant 4 × T**β**4 protein of the expected size (30.87 kDa) was produced following the induction of the bacterial cultures with isopropyl **β**-D-thiogalactoside (IPTG). The *E. coli*-derived 4 × *T*β**4 was purified by Ni-NTA resin, and its activities were examined with regard to both stimulating proliferation of the mice spleen cells *in vitro* and *in vivo* wound healing. The results demonstrate that these activities of the *E. coli*-derived recombinant 4 × T**β**4 were similar or even better than existing commercially obtained T**β**4. This production strategy therefore represents a potentially valuable approach for future commercial production of recombinant T**β**4.

## 1. Introduction

Thymosin *β*4 (T*β*4), a small acidic polypeptide (43-amino acid residues), was first discovered and isolated firstly from calf thymus [1], and encoding gene was mapped to the X chromosome [2]. T*β*4, which has a molecular weight of 4,982 Da and an isoelectric point of 5.1, is one of the most important thymosin in fraction 5, which participates in the regulation of thymus-dependent immune response [1]. 

T*β*4 is localized in the cytoplasm, shows a high affinity for G-actin, and serves as an G-actin-sequestering protein that regulates actin polymerization. It forms a 1 : 1 complex with monomeric G actin to maintain a dynamic equilibrium between G actin and F actin, preventing polymerization into actin filaments, supplies a pool of actin monomers when the cell needs filaments, and is thus critical for rapid reorganization of the cytoskeleton [2–6]. The amino acid residues of the T*β*4 protein that are critical for action binding were confirmed by chemical cross-linking and shown to correspond to a hydrophobic cluster consisting of residues (M6-I9-F12) in N-terminal *α*-helix, as well as K14 and K18 [7]. 

Several studies have suggested that T*β*4 contributes to various biological functions in different pathological stages and physiological processes, including the migration of epidermal cell and collagen, as well as formation of blood-vessel in the both wound healing [8, 9] and the induction of angiogenesis [3, 10, 11]. In particular, T*β*4 has been associated with healing of diabetic ulcers, bedsores, damaged corneas, and heart muscle injured during heart attacks [12, 13]. T*β*4 also plays important roles in the inhibition of inflammation [14, 15], as well as T-cell maturation, proliferation of cell and differentiation [13, 16].

T*β*4 is also present in tumor cells [2], and several research groups have found that aberrant expression of T*β*4 is associated with metastasis [10] and invasion of tumor cells [3], as seen in colorectal carcinoma [17, 18], gastrointestinal stromal tumors [2], breast cancer cells [19], melanoma, and lung tumors in mouse [10]. It has also been shown that T*β*4 promotes the growth of cancerous tumors by enhancing new blood-vessel formation [12]. However, other studies have resulted in contrary conclusions. For instance, the expression level of T*β*4 is significantly lower in multiple myeloma, and it has been suggested that T*β*4 can function to suppress the proliferation of tumor cells in myeloma development [3]. Moreover, another analysis concluded that overexpression of T*β*4 does not cause an increase in cell number in tumors in a transgenic strain of mice [12]. 

Although there is still considerable debate about the actions of T*β*4 in the context of tumor biology, it was concluded that “the clinical prospects for at least two thymosin proteins are finally looking brighter since first medical experiment at UCSF (University of California, San Francisco) more then [sic] a quarter-century” [12], among which, T*β*4 is one of the most promising prospects for clinical application; the other is thymosin *α*1.

In addition, T*β*4 is a promising molecular marker or therapeutic agent to serve the diagnosis and prognosis of certain diseases [2, 20], or to treat some common and frequently occurring conditions, and has been proposed to have value in the wound healing of diabetic ulcers, bedsores, and damaged corneas [9, 15]. 

In order to meet a potential increase in the clinical demand for T*β*4, the goal of this study was to develop a production pipeline for recombinant T*β*4. A DNA sequence, based on the T*β*4 coding sequences, was designed to generate a 4 × T*β*4 concatemer (four copies of the *T*β**4 gene arranged end to end in tandem, thereafter 4 × *Tβ*4), and two different bioactivities of resulting recombinant 4 × T*β*4 expressed in *Escherichia coli* were evaluated.

## 2. Materials and Methods

### 2.1. Synthesis of T*β*4 and Construction of the pET28a-6 × his-4 × T*β*4 Vector

Based on the T*β*4 amino acid sequence, a *T*β**4 gene was designed and synthesized [1], and DNA fragment of the *T*β**4 gene was subcloned into the pUC57 plasmid (termed pUC57-T*β*4). The pUC57-T*β*4 plasmid was digested with two combinations of restriction enzymes of *Spe *I/*Sac *I and *Xba *I/*Sac* I, and the resulting DNA was ligated into *Spe*I/*Sac*I sites of pUC57-T*β*4 to create pUC57-2×T*β*4. The pUC57-4 × T*β*4 containing four repeats of the *T*β**4 gene was also generated based on the isocaudamer property of *Spe* I and *Xba* I.

To facilitate the purification of the recombinant 4 × T*β*4 protein, a DNA sequence which is encoding six histidines was added at 5′ end of the 4 × *Tβ*4 concatemer by PCR technique using the primers of T*β*4F1 (5′-ggggatccatgcaccaccaccaccaccacggtaccatgtctagaatgtctga-3′) and T*β*4R1 (5′-ccgagctcttaactagtcataga-3′). The PCR product was gel purified and ligated into the plasmid pMD18 (Takara Biotechnology, Dalian, China) and termed pMD18-6 × his-4×T*β*4. Subsequently, the plasmids pMD18-6 × his-4 × T*β*4 and pET28a (Merck Millipore, Germany) were digested with *Bam *H I/*Sac* I, and the vector pET28a-6 × his-4 × T*β*4 was constructed following incubation with T_4_ DNA ligase (Takara Biotechnology, Dalian, China). The recombinant pET28a-6 × his-4 × T*β*4 was transformed into *E. coli* strain BL21 by a thermal impulse at 42°C for 90 seconds.

### 2.2. Confirmation of the *E. coli*-Derived 4 × T*β*4 Production

The BL21 cell strain harboring pET28a-6 × his-4 × T*β*4 was cultured in a 15 mL flask with 3 mL LB broth liquid medium (pH = 7.5) containing kanamycin (100 *μ*g/mL) in a shaker (220 rpm) at 37°C for overnight. A similar culture of the BL21 cell strain transformed with pET28a was also grown and used as a negative control. Both cultures (200 *μ*L) were added to 50 mL fresh LB medium with kanamycin (100 *μ*g/mL) and cultured to the OD_600_ = 0.5 in a 37°C shaker (220 rpm). Then IPTG was added to a 1 mM final concentration and incubated as above for 0 h to 8 h.

The BL21 cells from a 1 mL aliquot culture were collected by centrifugation at 8000 g for 10 min then resuspended in 10 *μ*L of the 1 × PBS buffer (140 mM NaCl, 2.7 mM KCl, 10 mM Na_2_HPO_4_, 1.8 mM KH_2_PO_4_, PH 7.4). The cells were then mixed with 10 *μ*L of 2 × SDS/PAGE sample buffer (120 mM Tris-HCl pH6.8, 20% glycerol, 4% SDS, 3%  *β*-mercaptoethanol, 0.02% bromophenol blue) and boiled in a water bath (100°C) for 5 min. The boiled samples were centrifuged at 8000 g for 5 min at 4°C, and 7 *μ*L supernatant from each sample was loaded a SDS-12% polyacrylamide gels and then subjected to electrophoresis in 1×Tris-Glycine Buffer (0.025 M Tris, 0.25 M glycine, 0.1% (w/v) SDS) at 100 volt at 4°C for 2 h. Duplicate gels were stained with 1% Coomassie Brilliant Blue (R250), another one, or electro-transferred to 0.22 *μ*m PVDF (Polyvinylidene Fluoride, Bio-Rad, USA) membrane using a Bio-Rad transfer equipment (35 volt at 4°C) for overnight for Western blotting analysis. A mouse anti-His-tag monoclonal antibody (Shanghai ImmunoGen Biological Technology, Shanghai, China) and an AP-conjugated goat anti-mouse antibody (Shanghai ImmunoGen Biological Technology, Shanghai, China) were, respectively, used as primary and second antibodies, respectively, to detect the expression of the recombinant 4 × T*β*4 protein.

### 2.3. *In Vitro* 4 × T*β*4 Stimulates Cell Proliferation

The cells harboring pET28a-6 × his-4 × T*β*4 were collected by centrifugation after IPTG (1 mM) induction at 37°C for 6 h, and the cells pellet was resuspended in PBS buffer (v : v = 1 : 5) and then sonicated at 200–300 W in an ice bath 6 times (pause/sonication = 10 s/10 s per time). The sonicated crude lysate was centrifuged under 4°C at 10 000 g for 30 min and the supernatant was discarded. The precipitated cells pellet (per gramme) was resuspended in 5 mL buffer B (8 M urea, 0.1 M sodium phosphate buffer, 0.01 M Tris-Cl, pH 8.0) and shaken gently at room temperature for approximately 30 min until the solution became translucent. The mixture was then mixed with Ni-NTA resin (Qiagen Gmbh, Germany) and loaded into a column. The flow-through from the column was collected. Nonbound proteins were collected by passing 20 mL buffer C (8 M urea, 0.1 M sodium phosphate buffer, 0.01 M Tris-Cl, pH 6.3), and the proteins adsorbed with Ni-NTA resin were eluted with 5 mL buffer E (8 M urea, 0.1 M sodium phosphate buffer, 0.01 M Tris-Cl, pH 4.5). 

The bioactivity of the *E. coli*-derived-4×T*β*4 protein in stimulating mice splenic lymphocyte proliferation was tested by a MTT-(3-(4,5-dimethylthiazol-2-yl)-2,5-diphenyl-2H-tetrazolium bromide) based method *in vitro *[21]. Spleen cells were isolated from 6-to 8-week-old Balb/c mice. The cells were collected by centrifugation at 1000 g at room temperature for 10 min, and the cells pellet was resuspended and diluted to 1 × 10^5^cells per mL in RPMI 1640 culture medium (Sigma-Aldrich, Shanghai, China).

A 100 *μ*L aliquot of the diluted spleen cells was added to each well of a 96-well plate. Samples of the *E. coli*-derived 4 × T*β*4 and commercially obtained T*β*4 protein (GL Biochem, Shanghai, China) were diluted to 250 ng/*μ*L in 1 × PBS buffer, and a 100 *μ*L of the diluted 4 × T*β*4 was added to each well (six replications per treatment). Aliquots (100 *μ*L) of the diluted commercial T*β*4 protein and 1 × PBS buffer were used as positive and negative controls, respectively. The 96-well plate was incubated in a cell culture incubator at 5% CO_2_ at 37°C for 24 h. After incubation, 10 *μ*L of MTT reagents was added into each well. Then the plate was then returned to the cell culture incubator for an additional 4 h. The cells in the 96-well plate were periodically examined with a CKX31 inverted microscope (Olympus, Watford, Herts, UK). When a purple precipitate was clearly visible, 100 *μ*L of dimethyl sulfoxide (DMSO) was added and the plate was then gently swirled and placed in the dark at room temperature for 15 min. The absorbance was read at 570 nm with a Microplate Reader (Bio-Tek, Power Wave XS, USA). The mouse splenic lymphocyte proliferation rate was calculated according to formula: proliferation rate = (OD_test_ − OD_control_)/OD_test_.

### 2.4. *In Vivo* 4 × T*β*4 Promotes Wound Healing

Three full-thickness punch wounds (5 mm diameter) were made on dorsal surfaces of each Balb/c mouse (6- to 8-week-old, *n* = 6) (Shanghai SALES Laboratory Animal, Shanghai, China), as previously described [22, 23]. Both the *E. coli*-derived 4 × T*β*4 and commercial T*β*4 protein were diluted to 0.1 *μ*g/*μ*L using 0.9% (w/v) physiological saline. Three punch wounds per mouse were topically treated with the 4 × T*β*4, the commercial T*β*4 protein and 0.9% (w/v) physiological saline, respectively, 24 h and 48 h after wounding.

In the first experimental group of three mice, keratinocyte migration was determined by measuring the lengths of the epidermal tongues from wound edges with a vernier caliper (Endura-Greenlee Tools, E0531, Shanghai, China) from 2 to 10 days after application treatment, and the percentage wound of closure was calculated by formula: percentage wound closure = distance of migrated keratinocytes from the wound edge/total wound width × 100% [23].

A second experiment group of three mice was used to examine reepithelialization and vessel counts (angiogenesis) in the wound bed. The mice were euthanized on day 8 after wounding, and the skin tissues were taken from wounds, fixed in 4% (v/v) formalin buffer, and then embedded in paraffin after dehydration in series of increasing ethanol concentrations (75%, 85%, 95%, and 100%). The embedded skin tissue samples were sliced into 5 *μ*m thick sections using a microtome (Leica, RM2200), and the sections from middle of the wounds were mounted on glass slides and then were stained with hematoxylin and eosin (Shanghai Dingjie Biotechnology Company, Shanghai, China) after paraffin removal, and the samples were evaluated using a microscope (Olympus BX51, Japan). 

Histological sections were used to measure the vessel counts as previously described by Malinda et al. [22]. Vessel counts were determined by first identifying vascular spaces by their endothelial lining. All such vessels in the wound bed were counted, including those at the junction of the dermis and the subcutis, since angiogenesis in the wounds occurs to a great extent from these vessels. The vessel counts were taken from mean value of 10 view fields (40×) per treatment. Statistical analysis pf the data was performed using Statistical Product and Service Solutions (SPSS) with a *P* value <0.05 [24]. All the above analyses were performed using double blind trials.

## 3. Results

### 3.1. Synthesis of T*β*4 Gene and Construction of pET28a-6 × his-4 × T*β*4

According to amino acid sequence of *Tβ*4 protein, a 168 bp of *Tβ*4 gene was designed and synthesized, which includes restriction enzyme sites (underlined), protection bases, and initiation codon (ATG) and the termination codon (TAA) (bold), as shown below:


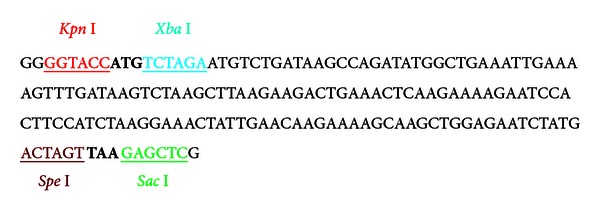


**Figure 1:**
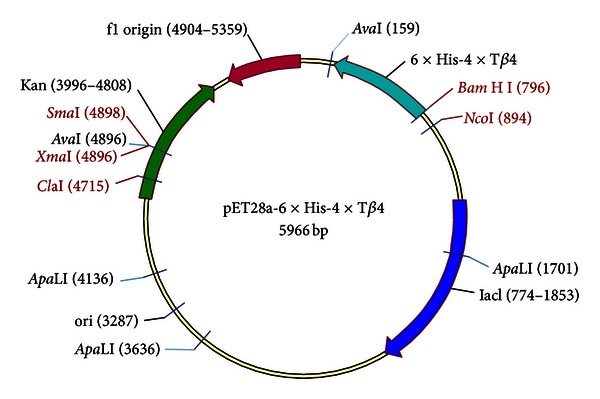
Diagramme of pET28a-6 × his-4 × T*β*4. lac I, lac repressor coding gene; Kan: kanamycin coding gene; 6 × his, a DNA sequence encoding six histidines; 4 × *Tβ*4, four copies of a DNA sequence which encode *Tβ*4 arranged in tandem; origin, DNA sequence of pBR322 origin of replication; f1 origin, DNA sequence of phage f1 origin of replication.

The 4 × T*β*4 was created using isocaudamer property of *Xba* I and *Spe* I, and a short DNA sequence (18 base pair) encoding six histidines was introduced at 5′-end of the 4 × *Tβ*4 sequence by PCR, yielding a DNA sequence of the 6 × his-4 × *T*β*4* (619 bp). The DNA fragment was subsequently inserted into the *Bam* H I and *Sac* I sites in the pET28a plasmid to generate pET28a-6 × his-4 × T*β*4 (Figure 1). 

### 3.2. Induced Expression of 4 × T*β*4 Protein

**Figure 2:**
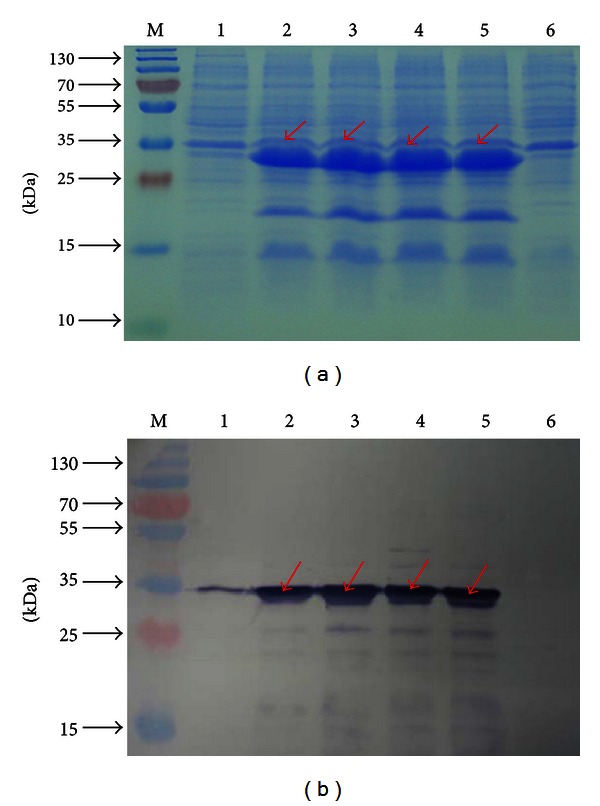
(a) Expression of recombinant 4 × T*β*4 in BL21 cells at different induction times. Lane M, middle-molecular mass protein markers; lanes from 1 to 5: induced expression of the recombinant 4 × T*β*4 by IPTG (1 mM) at 0, 2, 4, 6, and 8 hs, respectively; lane 6: induced BL21 containing pET28a by IPTG (1 mM) for 6 hs. Red arrows indicate specific band of *E. coli*-derived 6 × his-×T*β*4. (b) Western blot analysis of the *E. coli*-derived 6 × his-4 × T*β*4. Lane M: middle-molecular-mass protein markers; lanes from 1 to 5: BL21 harboring pET28a-6 × his-4 × T*β*4 after IPTG (1 mM) induction at 37°C for 0, 2, 4, 6, and 8 h, respectively; lane 6: induction of expression of the BL21 containing pET28a by IPTG (1 mM) at 37°C for 6 h. Red arrows indicate the specific hybridization signal for the *E. coli*-derived 6 × his-×T*β*4.

To confirm the induced expression of 4 × T*β*4 protein in* E. coli*, rude proteins extracted from BL 21 cell pellets were separated by SDS/PAGE gels, and the expected 30.87 kDa recombinant 4 × T*β*4 protein was monitored following IPTG induction for 0 h to 8 h in BL 21 harboring pET28a-6 × his-4 × T*β*4 the expected band was detected at all time increments other than 0 h (Figure 2(a)). In order to further confirm the presence of 4 × T*β*4 protein, the mouse anti-His-tag was used as a primary antibody in a Western blot analysis of the protein extracts. The expected hybridization signal at 30.87 kDa was detected at different induction times from 0 h to 8 h in BL 21 harboring pET28a-6 × his-4 × T*β*4; however the signal was much weaker in 0 h sample. As expected, no immunoreactive band corresponding 4 × T*β*4 was detected in the negative control BL21 samples form cultures transformed with pET28a (Figure 2(b)).

### 3.3. *In Vitro* 4 × T*β*4 Stimulates Cell Proliferation

**Figure 3:**
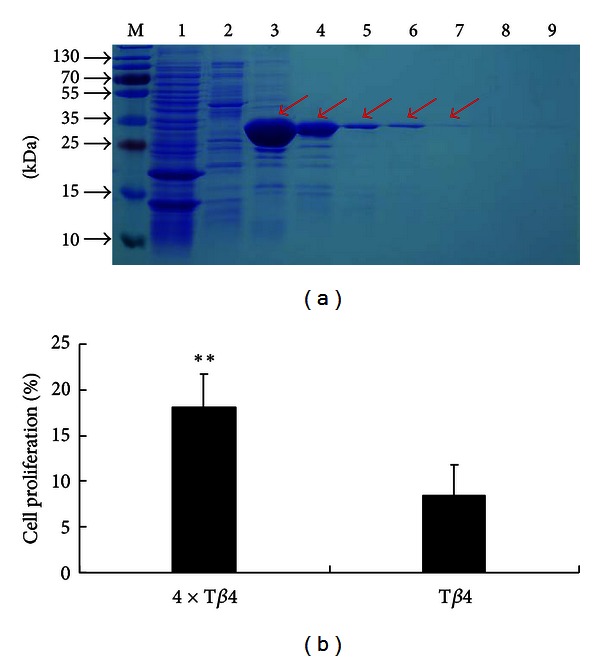
(a) Purification of the *E. coli*-derived 4 × T*β*4. Lane M: middle molecular mass protein markers; lane 1: unbound proteins were eluted using 1×Ni-NTA buffer B; lane 2: nonspecific proteins were eluted using 1 × Ni-NTA buffer C; lanes from 3 to 9: 4 × T*β*4 was eluted using 1 × Ni-NTA buffer E. Red arrows indicate the specific band of *E. coli*-derived 4 × T*β*4 on SDS/PAGE gel. (b) Assay of mice splenic lymphocyte proliferation using MTT method. 4 × T*β*4: purified 4 × T*β*4 protein extracted from BL21 (*E. coli*) stimulate, mice splenic lymphocyte proliferation; T*β*4: commercial T*β*4 protein (GL Biochem) stimulates mice splenic lymphocyte proliferation.

In order to confirm the bioactivity of the recombinant 4 × T*β*4, the* E. coli*-derived 4 × T*β*4 protein was purified by the Ni-NTA resin and examined by SDS-PAGE gel. The results showed that both unbound and nonspecific bound proteins were eluted from the column using 1×Ni-NTA buffer B and C, respectively; however the 4 × T*β*4 protein with 6 × his fusion was successfully obtained by elution of bound proteins from the Ni-NTA resin (Figure 3(a)).

Both *E. coli*-derived 4 × T*β*4 proteins and commercial T*β*4 were subsequently diluted to 250 ng/*μ*L in 1×PBS buffer and then used to in a MTT assay. The MTT results showed that the *E. coli*-derived 4 × T*β*4 promotes mice lymphocyte proliferation (18.13 ± 3.65%), to a significantly higher degree than the commercial T*β*4 (8.49 ± 3.32%) (Figure 3(b), *P* < 0.01). 

### 3.4. *In Vivo* 4 × T*β*4 Promotes Wound Healing

**Figure 4:**
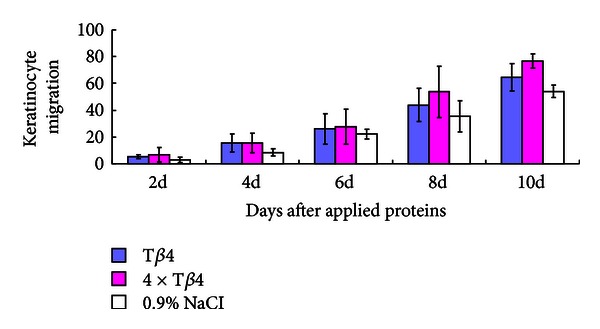
Change of keratinocyte migration in wound bed at different times after application treatment. T*β*4: wounds of the applied standard T*β*4 purchased from GL Biochem; 4 × T*β*4 (BL21): wounds of the applied *E. coli*-derived 4 × T*β*4; NaCl: wounds of the applied 0.9% physiological saline; 2 d to 10 d: days after application.

The full thickness cutaneous mouse wound model was employed to examine the efficiency of the *E. coli*-derived 4 × T*β*4 in wound healing. Keratinocyte migration was determined by measuring the lengths of the epidermal tongues from the wound edges. The results showed that reepithelialization is increased in all treatments and that rate was higher following treatment with *E. coli*-derived-4×T*β*4 than that with both positive control (commercial T*β*4) and negative control (0.9% physiological saline). However, the difference is insignificant between treatments from 2 days to 6 days after application treatment. Keratinocyte migration was significantly higher following treatment with the* E. coli*-derived 4 × T*β*4 than the negative control from day 8, and the percentage of wound closure reached 76.72 ± 5.54%. On day 10, the closure was significantly higher (*P* < 0.01) than the negative control (54.05 ± 4.55%) (Figure 4, see Supplementary Figure  1 available online at http://dx.doi.org/10.1155/2013/241721); however, there was no significant difference between treatments with the *E. coli*-derived 4 × T*β*4 and commercial T*β*4. 

**Figure 5:**
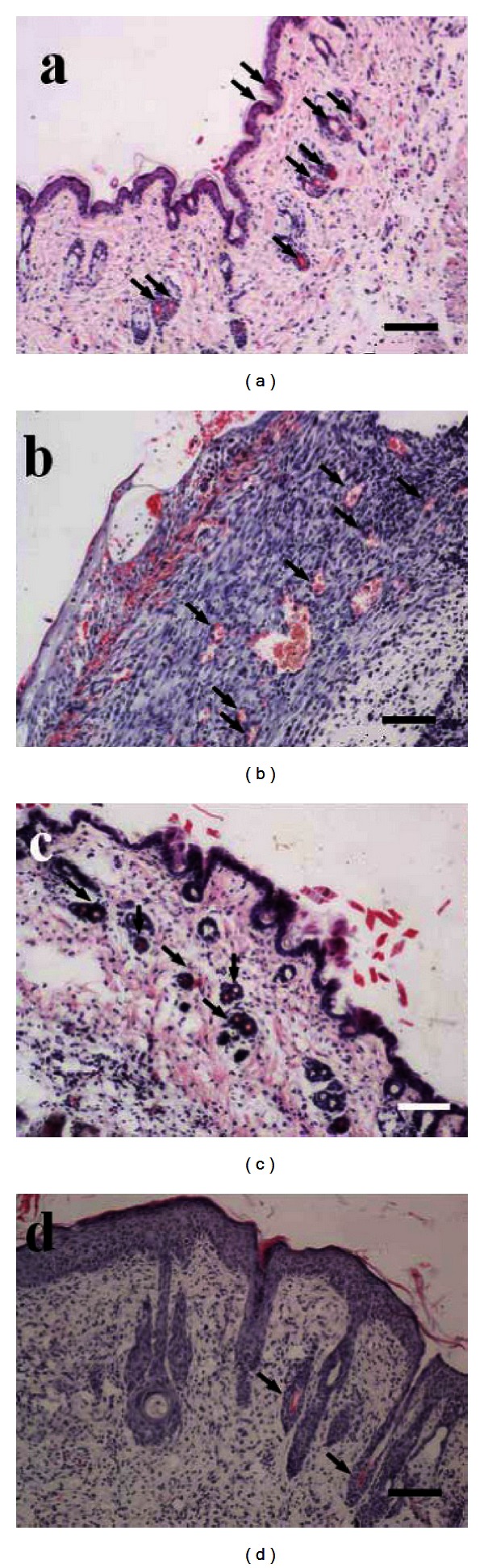
Histological sections of reepithelialization and angiogenesis at day 8 after wounding. Arrows indicate the formation of blood vessel in the wound bed. (a) The newly formed blood vessels and reepithelialization of the wound epidermis in wound location by topical treatment with 5 *μ*g 4 × T*β*4 (dissolved in 50 *μ*L of the 0.9% physiological saline). (b) The newly formed blood vessels and reepithelialization of the wound epidermis in the wound location by topical treatment with 5 *μ*g T*β*4 (dissolved in 50 *μ*L of the 0.9% physiological saline). (c) Treatment with 0.9% physiological saline. (d) Normal mouse skin tissues (bar = 100 *μ*m).

The *E. coli*-derived 4 × T*β*4 also promoted an increase in the number of blood vessels in wound bed, as determined by observing tissue sections on day 8 after wounding. The blood vessel number (10.33 ± 0.58 vessel number/9 × 10^4^ *μ*m^2^, *P* < 0.01) was significantly greater than that of the negative control (5.00 ± 1.00 vessel number/9 × 10^4^ *μ*m^2^); however, there was no significant difference between the *E. coli*-derived 4 × T*β*4 and the commercial T*β*4 (10.00 ± 1.00 vessel number/9 × 10^4^ *μ*m^2^) samples (Figure 5).

## 4. Discussion 

The T*β*4 protein has been proposed as one of the two most promising thymosins (the another being thymosin *α*1) for future clinical applications [12]. Several research groups have suggested that T*β*4 protein has the potential to cure or prevent many diseases associated with the immune system and can be used to treat inflammation [14, 15] and tumors [2, 3] and promote wound healing [8]. The number of patients with diseases such as diabetes is gradually rising in conjunction with an aging population and suboptimal life style often resulted in diabetic ulcers and bedsores. T*β*4 protein is therefore likely to have increasing importance as a therapeutic agent, which in turn suggests that there will be an increasing demand for T*β*4 production. Currently, the T*β*4 is produced commercially by two approaches: extraction from animal thymus [1] and chemical synthesis [25], but there are challenges with meeting clinic demand due to risks of zoonosis and higher production costs [25, 26]. 

We therefore sought to produce T*β*4 protein via a genetic engineering approach, and a gene encoding T*β*4 was designed and synthesized. To enhance expression and facilitate purification, a recombinant *T*β*4* concatemer protein fused to a histidine tag was expressed in and purified from *E. coli* cultures. 

Several studies have revealed that T*β*4 functions by binding to G-actin [3–6], and the bioactivity of T*β*4 protein was related to its structure and amino acid components [7, 27]. As of yet, we have no evidence that the *E. coli*-derived 4 × T*β*4 has similar binding characteristics equal to the commercial T*β*4, which is generated by chemosynthesis or extraction of animal thymuses. However, the results showed that the *E. coli*-4 × T*β*4 protein promotes cell proliferation *in vitro* (Figure 3(b)), as well as wound healing and vessel increase *in vivo *(Figures 4 and 5), and its capabilities in these regards are at least similar, or even better than commercial T*β*4. Further research focuses on producing T*β*4 using other expression systems including yeast and plant [28–32].

## Supplementary Material

Supplemental Figure: Wound healing of the mice at different times post wounding. The 1 day to 10 day on the plate are days of post wounding, respectively. The A, B and C are wounds treaded by commercial T**β**4 protein, *E. coli*-derived recombinant 4 × T**β**4 protein and 0.9% physiological saline, respectively.
