# Supplementary material for: The Escherichia coli-Derived Thymosin β4 Concatemer Promotes Cell Proliferation and Healing Wound in Mice
Source: Biomed Res Int. 2013 May 19;2013:241721. doi: 10.1155/2013/241721 (PMC3671520; doi:10.1155/2013/241721)
Supplement: Supplementary file 1 — Supplemental Figure: Wound healing of the mice at different times post wounding. The 1 day to 10 day on the plate are days of post wounding, respectively. The A, B and C are wounds treaded by commercial Tβ4 protein, E. coli-derived recombinant 4 × Tβ4 protein and 0.9% physiological saline, respectively. [file 241721.f1.pdf]

Supplemental gross photographs of the mice

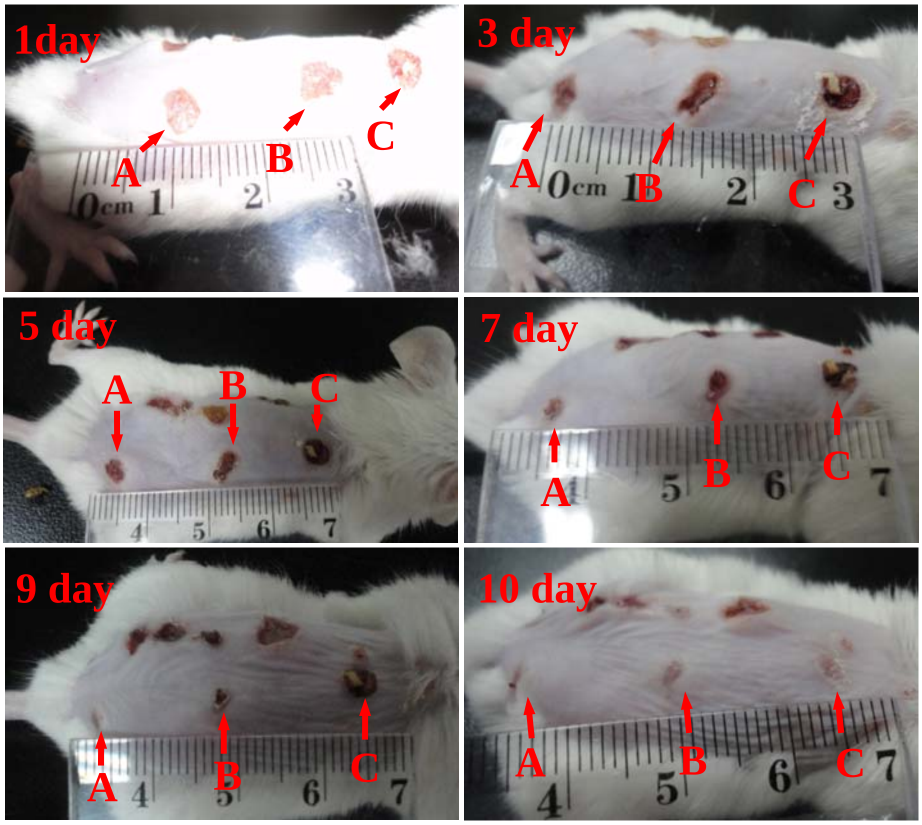

Supplemental Figure 1. wound healing of the mice at different times post wounding

The 1 day to 10 day on the plate are days of post wounding, respectively. The A, B and C are wounds treaded by commercial Tβ4 protein, *E.coli*-derived recombinant 4×Tβ4 protein and 0.9% physiological saline, respectively.
